# An Improved Real-Time R-Wave Detection Efficient Algorithm in Exercise ECG Signal Analysis

**DOI:** 10.1155/2020/8868685

**Published:** 2020-07-29

**Authors:** Zhou Zhang, Zeyu Li, Zhangyong Li

**Affiliations:** ^1^Research Centre of Biomedical Engineering, Chongqing University of Posts and Telecommunications, Chongqing 400065, China; ^2^Chongqing Engineering Research Center of Medical Electronics and Information Technology, Chongqing University of Posts and Telecommunications, Chongqing 400065, China

## Abstract

R-wave detection is a prerequisite for the extraction and recognition of ECG signal feature parameters. In the analysis and diagnosis of exercise electrocardiograms, accurate and real-time detection of QRS complexes is very important for the prevention and monitoring of heart disease. This paper proposes a lightweight R-wave real-time detection method for exercise ECG signals. After real-time denoising of the exercise ECG signal, the median line is used to correct the baseline, and the first-order difference processing is performed on the differential square signal. Max-Min Threshold (MMT) is used to realize real-time R-wave detection of the exercise ECG signal. The abovementioned method was verified by using the measured data in the MIT-BIH ECG database of the Massachusetts Institute of Technology and the exercise plate experiment. The R-wave detection rates were 99.93% and 99.98%, respectively. Experimental results show that this method has high accuracy and low computational complexity and is suitable for wearable devices and motion process monitoring.

## 1. Introduction

ECG (Electrocardiogram) represents the myocardial electrical activity of the heart. ECG signals play an important role in the diagnosis of cardiovascular diseases, such as arrhythmia, hypertension, or ischemic heart disease. ECG recording used to be a time-consuming process that required an on-site cardiologist to detect and diagnose various types of heart disease. Today, ECG signals can be recorded using mobile ECG sensors, such as Shimmer sensor or Alivecor sensor [1]. These sensors are not only easy to use but also economical and efficient to obtain ECG signals. However, these sensors mainly use only 1 or 2 leads (usually lead I or lead II) for recording, rather than all standard 12-lead, resulting in a poor detection performance of some QRS detection algorithms. Therefore, real-time detection and anomaly analysis of the QRS waveform is a challenging task [2].

QRS detection has become a research topic in the field of intelligent ECG detection for more than 30 years. In the meantime, researchers have developed a number of algorithms, for example, based on the digital filter [3], wavelet transform [4, 5], neural network [6–8], image segmentation [9], and so on. Tang et al. proposed a parallel incremental modulator architecture with local maximum point and local minimum point algorithms to detect QRS and PT waves [10]; Kalidas and Tamil proposed an online QRS detector algorithm using stationary wavelet transform (SWT) for real-time heartbeat detection from a single lead ECG signal [4]; Muhammad et al. proposed the use of transient phasor transformation to study the location of characteristic points (reference points) of ECG signals [11]. However, most of the studies are only applicable to the static ECG signal for a long time, and the processing ability of motion interference is not strong. Moreover, the abovementioned methods require additional process steps, such as training, setting, and predicting model parameters, when detecting R-waves, which increases the complexity of calculation load and computing cost [12].

The R-wave in the QRS waveform plays an important role in the diagnosis of arrhythmia and the recognition of heart rate variability. Due to the rise of wearable devices, the noninvasive exercise tablet experiment (stress test) and the high prediction of coronary heart disease and other diseases, wearable devices, and exercise tablet experiment have gradually become popular in the detection of the cardiovascular function. However, mobile devices are used to detect the ECG activity of patients in their daily life, rather than in the isolated hospital environment. As a result, various electrical signal noises in the environment will seriously interfere with ECG signals. Exercise ECG detection will cause the ECG signals of patients to be interfered by the electrical signals generated by human muscle movements during exercise, which will lead to a sharp increase in detection Windows, thus increasing the possibility of detecting errors and detecting omissions by the traditional R-wave detection algorithm [13].

Aiming at the characteristics of difficulty in real-time detection and abnormal analysis of single lead of motion electrocardiogram, large motion interference, and high computing cost, this paper proposes a lightweight adaptive Max-Min Threshold (MMT) algorithm for R-wave detection of a motion ECG signal, which is an optimization of the differential threshold method for R-wave detection. Compared with the traditional R-wave detection algorithm, the algorithm proposed in this paper has lower operation cost, higher anti-environment interference and anti-motion interference ability, and is suitable for the medium and long-term exercise ECG detection on the mobile ECG sensor. In order to determine the detection efficiency, the algorithm performed the exercise ECG acquisition and real-time R-wave detection on the exercise plate. Meanwhile, the ECG data from the MIT-BIH arrhythmia database were used for R-wave detection.

## 2. Lightweight R-Wave Detection Method

The detection of R-waves in ECG signals presents many challenges, such as EMG interference [14], power-frequency interference, and baseline drift, which can affect signal primitiveness. These noises are the difficulties in the automatic detection of ECG signals. Due to the large body swing and electrode friction caused by movement, the noise is particularly prominent in the motion ECG signals.

**Figure 1:**
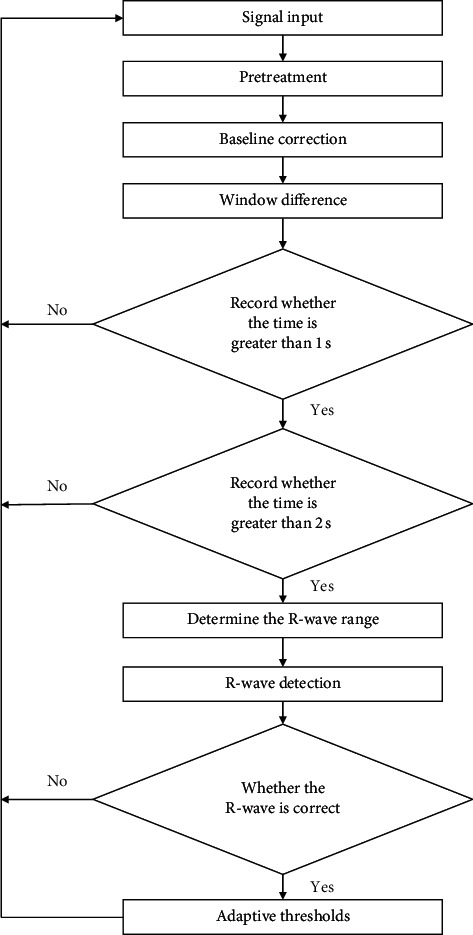
Algorithm flowchart.

This paper proposes an adaptive MMT difference algorithm to detect R-wave, which includes the following steps: the whole algorithm process of preprocessing baseline correction for R-wave detection is shown in Figure 1.

Firstly, in order to eliminate the noise embedded in the ECG signal and enhance the ECG signal, digital filters with appropriate parameters and adaptive filters are usually used to eliminate the noise [15]. In this paper, an FIR filter and Notch filter are used to eliminate EMG interference and power-frequency interference in the ECG signal. This process has a large amount of ECG processing capacity and a lot of detail processing, but it does not affect the detection of R-wave. In addition, the calculation cost of the filtering process is lower and the space occupancy is less.

Secondly, a baseline drift can greatly interfere with overall R-wave detection, for which a sliding window is used to overcome the baseline drift. The ECG baseline was extracted from the ECG through the sliding window, and the difference between the original signal and the ECG baseline was calculated to obtain the ECG signal after the baseline was more positive.

Finally, the amplitude of the R-wave varies from person to person, and the amplitude of the R-wave varies greatly from person to person at different times. Therefore, it is very important to find the appropriate threshold. In this paper, through adaptive multithreshold to cope with different scenarios, different populations, and different collection patterns, the R-wave is accurately calculated.

### 2.1. Pretreatment

Based on the power-frequency interference and myoelectric interference existing in the ECG acquisition process, the R-wave detection is greatly affected, so pretreatment is needed to eliminate the corresponding interference. This section will discuss the power-frequency interference and myoelectric interference in the ECG acquisition process and how to remove the noise.

#### 2.1.1. Power-Frequency Interference

Frequency interference at 50 Hz is usually eliminated before further analysis and processing of the signal [16]. In addition, the basic principle of an adaptive Notch filter is a center frequency of an orthogonal signal as the reference signal, using the linear combination of the orthogonal signal tracking the input signal, and through every step of the residual, continuously adjust the weights of linear combination, so as to make the input signal related to the reference signal linear part of the separation, to achieve the effect of a narrow-band filter.

**Figure 2:**
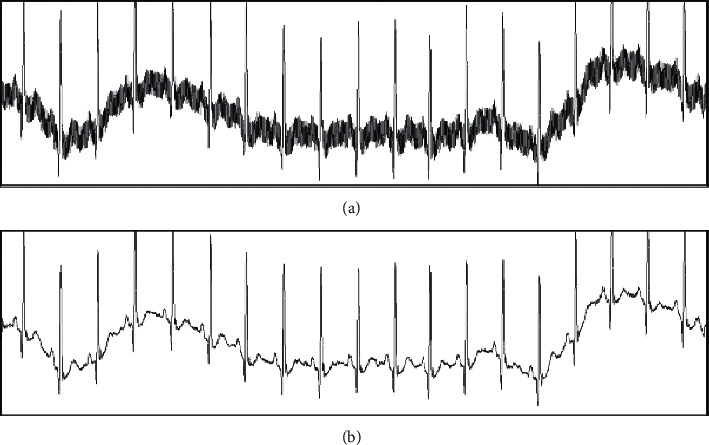
Notch filter effect. (a) Before the filtering. (b) After the filtering.

In this paper, a Notch filter is used to filter the received ECG signal, so as to eliminate the capacitance and electrode lead loop distributed in the human body from 50 Hz power-frequency interference such as power-frequency electricity and magnetic field. According to the Notch filter selected, its filtering effect is shown in Figure 2.

#### 2.1.2. EMG Interference

Because FIR and IIR filters show maximum signal-to-noise ratio improvement when used to eliminate interference, these simple filters are commonly used for ECG signal noise reduction [17].

A finite impulse response (FIR) filter is to perform weighted and average processing on *N* sampled data, in which the input signal is temporal and changes with the change of time. The final output of the FIR filter is the input at each moment multiplied by the corresponding weight (coefficient), then superimposed, and finally, output. The difference equation can be expressed as follows:

(1) $$\begin{array}{c} y\left(n\right)=\sum_{i=0}^{N-1}a_{i}x\left(n-i\right). \end{array}$$

**Figure 3:**
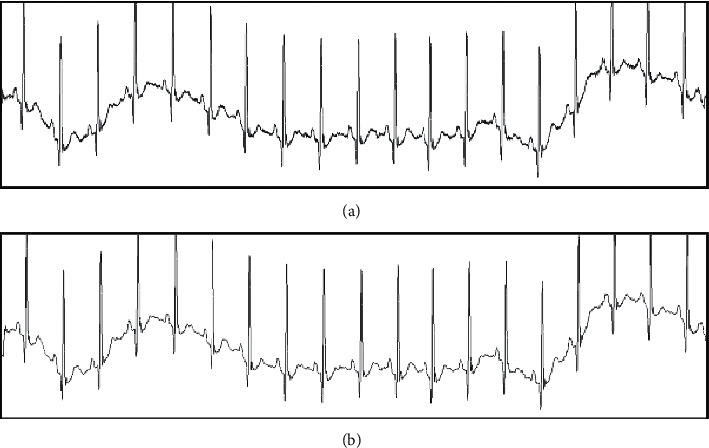
FIR filter effect. (a) Before the filtering. (b) After the filtering.

The low pass filter of an FIR is adopted to eliminate the noise greater than 100 Hz which does not belong to the range of the ECG signal. The filter is of order 40, with a sensitivity factor of 40, a sampling frequency (FS) of 500 Hz, a passband frequency (*F*pass) of 6, a stopband frequency (*F*stop) of 100, a passband waste (*W*pass) of 3 db, and a stopband waste (*W*stop) of 1 db. According to the optimal approximation method of FIR and other ripples selected, its filtering effect is shown in Figure 3.

### 2.2. Baseline Correction

Baseline drift often occurs in the motion ECG signal, especially when the subject swings too much and the lead line wobbles more, resulting in a very serious baseline drift. Because median filtering can effectively discard outliers while retaining relevant information, median filtering has been widely used as a postprocessing operator in different fields [18] and is widely used in biomedical signal processing [19].

In this paper, the ECG signal end is wrapped by a large sliding window, and the median amplitude of the ECG data in the window is calculated as the baseline drift value of the middle position of the window. The baseline correction can be completed by subtracting the ECG amplitude from the baseline drift value. The algorithm of its window size *W* and ECG amplitude *Y* after removing baseline drift is as follows:

(2) $$\begin{array}{c} W=fs*\text{time }\left(+1\right),\\ Y\left(\text{index}-\frac{W}{2}\right)=X\left(\text{index}-\frac{W}{2}\right)-X\left(m\right), \end{array}$$

where *fs* is the ECG signal sampling rate, time is the time length, index is the index value of the current real-time ECG record, and *m* meets

(3) $$\begin{array}{c} P\left(\frac{\text{count }\left(x\left(i\right)\leq x\left(m\right)\right)}{W}\right)=\frac{1}{2}, \end{array}$$

where the value range of *I* is (index − *W*) ≤ *i* ≤ index.

Window size *W* is an odd-numbered window, and its window should contain at least 0.6 s of sample data, which is helpful to calculate the baseline of the ECG signal.

### 2.3. R-Wave Detection

The detection process of the R-wave mainly includes window difference, initial threshold calculation, MMT detection of the R-wave, error correction, and adaptive threshold.

#### 2.3.1. Window Difference

Signal differential algorithm is the first step to detect the R-wave. In this paper, a sliding window is used to wrap the differential data of the ECG signal, which can effectively reduce the memory consumption and improve the detection efficiency of real-time ECG. The difference amplitude *Y* algorithm is as follows:

(4) $$\begin{array}{c} \text{Temp}=X\left(n+2\right)-X\left(n\right),\\ Y\left(n\right)=\left\{\begin{array}{cc} -{\text{Temp}}^{2}, & \text{Temp}<0,\\ {\text{Temp}}^{2}, & \text{Temp}>0, \end{array}\right. \end{array}$$

where *X* represents the ECG signal processed by using a Notch filter (Section 2.1.1) and FIR filter (Section 2.1.2).

In this paper, a window of size 3 is set up to record the difference amplitude, which is represented by *Y*(*n* − 1), *Y*(*n*), and *Y*(*n*+1) in the follow-up, where *Y*(*n*+1) represents the largest difference amplitude that can be obtained.

#### 2.3.2. Calculation of Initial Threshold

In this paper, three thresholds are adopted to determine the position of the R-wave: the maximum threshold *T*_max_ of first-order difference, the minimum threshold *T*_min_ of first-order difference, and the threshold *T*_*R*_ of ECG amplitude. The initial process of the three thresholds is as follows:

(5) $$\begin{array}{c} T_{R}=X_{\max}*{\text{coef}}_{1},\\ T_{\max}=Y_{\max}*{\text{coef}}_{2},\\ T_{\min}=Y_{\min}*{\text{coef}}_{2}, \end{array}$$

where *X*_max_ is the maximum value of ECG after processing and *Y*_max_ and *Y*_min_ are the maximum and minimum values of differential signals, respectively.

Due to the characteristics of the finite unit impulse response (FIR) filter and adaptive Notch filter (Notch), the ECG signal 1s before recording should not be considered when calculating the threshold value.

#### 2.3.3. MMT Detects R-Waves

According to the maximum and minimum difference thresholds *T*_max_ and *T*_min_ obtained in Section 2.3.2, the maximum point in the range of *Y*(*n*) > *T*_max_ amplitude of differential signals in a continuous period of time was calculated through the continuously input ECG signals, and the index *S*_1_ of this point was recorded. The minimum point in the range of differential signal amplitude *Y*(*n*) < *T*_min_ in a continuous period of time is calculated, and the index *S*_2_ of this point is recorded. When *S*_1_ < *S*_2_ and the (*S*_1_, *S*_2_) range is within 0.2 ms, the maximum value *X*(*n*)_max_ of ECG after processing is calculated in the index range of point *S*_1_ and *S*_2_. When *X*(*n*)_max_ > *T*_*R*_, this point is considered to be point *R*.

(6) $$\begin{array}{c} \left\{\begin{array}{c} Y\left(n_{1}-1\right)<Y\left(n_{1}\right)\\ Y\left(n_{1}\right)>Y\left(n_{1}+1\right)\\ Y\left(n_{1}\right)>T_{\max} \end{array}\right.⟶S_{1}=n_{1},\\ \left\{\begin{array}{c} Y\left(n_{2}-1\right)<Y\left(n_{2}\right)\\ Y\left(n_{2}\right)>Y\left(n_{2}+1\right)\\ Y\left(n_{2}\right)<T_{\min} \end{array}\right.⟶S_{2}=n_{2},\\ \left\{\begin{array}{c} X\left(n-1\right)<X\left(n\right)\\ X\left(n\right)>X\left(n-1\right)\\ X\left(n\right)>T_{R} \end{array}\right.⟶R_{\text{pos}}=n.\; \end{array}$$

#### 2.3.4. Error Correction

In the detection of R-wave, some interference may lead to multiple detection and missed detection of the R-wave. In this paper, the error correction of the R-wave is carried out by the following methods. The index difference between the R-wave position and the previous R-wave position Dif is recorded, and it is compared with the previous index difference Last_Dif.

If Dif > 1.66*∗Last_Dif*, there may be a missed judgment between this R-wave and the previous R-wave. At this time, the threshold value of reduction amplitude is 90% of the original threshold value for redetection. If Dif < 0.6*∗Last_Dif*, the gap between the point and the previous R-wave is too small, which is considered as a misjudgment.

#### 2.3.5. Adaptive Threshold

According to the amplitude *D*_*S*_1__ and *D*_*S*_2__ corresponding to the index *S*_1_ and the index *S*_2_, as well as the amplitude *R*_*R*_ of point *R*, the three thresholds *T*_*R*_, *T*_max_, and *T*_min_ are updated adaptively according to the following formula:

(7) $$\begin{array}{c} T_{R}=T_{R}*\text{Rate}+R_{R}*\text{coef}*\left(1-\text{Rate}\right),\\ T_{\max}=T_{\max}*\text{Rate}+D_{S_{1}}*\text{coef}*\left(1-\text{Rate}\right),\\ T_{\min}=T_{\min}*\text{Rate}+D_{S_{2}}*\text{coef}*\left(1-\text{Rate}\right). \end{array}$$

Due to the difference in the amplitude of the R-wave in time, it is necessary to judge the amplitude of the current measured R-wave once in the abovementioned formula. If the amplitude of the R-wave is in the state of increasing (or decreasing) for two consecutive times, the adaptive updating of the threshold needs to be stopped.

## 3. Results and Discussion

### 3.1. Exercise ECG Data

**Figure 4:**
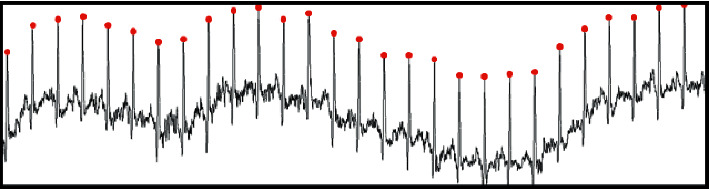
Exercise ECG real-time R-wave detection.

A panel exercise test was performed on 10 test subjects using disposable button electrodes. The heart rate of the subject was increased through exercise, the V1 and V2 leads of the subject are recorded, and the R-wave of the ECG waveform of the subject is detected and displayed in real-time, as shown in Figure 4.

It can be seen from Table 1 that this algorithm has a good performance in 10 subjects of different genders, and it can effectively monitor and recognize the R-waves of a total of 8619 ECG waveforms of 10 subjects. When the maximum heart rate was reached (195-age) [20], the body swing amplitude of the subjects reached the maximum, and the ECG signal received the maximum interference. This algorithm also had a good performance, and the R-wave detection results were accurate. At the same time, the memory utilization is low in the detection process.

### 3.2. MIT-BIH Public Database

The MIT-BIH arrhythmia database contains 48 1/2 hours of excerpts from two-channel dynamic ECG recordings. The records are digitized with 360 samples per second per channel, with an 11-bit resolution in the 10 mV range [21].The ANSI/AAMI/ISO EC57 : 1998/(*R*) 2008 standard states that the QRS detection algorithm must provide statistical reports from the MIT-BIH arrhythmia database [22].

In order to evaluate the performance of the proposed algorithm, common detector performance measurements are applied and defined as follows [23].

Sensitivity (Se) represents the percentage of events detected:

(8) $$\begin{array}{c} \text{Se}=\frac{\text{TP}}{\text{TP}+\text{FN}}\times 100\%. \end{array}$$

Positive prediction (+*P*) represents the score of the test, that is, the event:

(9) $$\begin{array}{c} +P=\frac{\text{TP}}{\text{TP}+\text{FP}}\times 100\%, \end{array}$$

where TP is the number of true positive beats (correct detection), FN is the number of false negative beats (false detection), and FP is the number of false positive beats (missed detection) [10].

As can be seen from Table 2, this algorithm successfully detected 47,398 R-waveforms in 47,498 QRS-waveforms, indicating that this algorithm can correctly detect the vast majority of R-waveforms. In addition, Table 3 shows a quantitative comparison of the algorithms presented in this paper with those proposed by Pandit and Lai. The values of Se and +*P* in this paper reach 99.70% and 99.93%, while Pandit's and Lai's algorithms are 99.62% and 99.67% and 99.69% and 99.63%, respectively. The algorithms of Pandit and Lai are both R-wave recognition algorithms based on differential thresholds. In the R-wave detection results, the method of this paper has improved by 0.05% and 0.3% on average.

## 4. Conclusions

This paper presents a lightweight adaptive MMT ECG signal R-wave detection algorithm. After denoising the ECG signal and correcting the baseline, the algorithm performs first-order difference processing, detects the R-wave through the maximum and minimum difference threshold, and updates the threshold according to the index information of the R-wave. The algorithm of R-wave detection in an athletic flat test is in good condition, and in the MIT/BIH database of 21 ECG data detection, through comparing with Pandit algorithm and Lai algorithm, the presented method of R-wave identification not only is of high sensitivity (Se) and high positive predictive (+*P*) but also has advantages in terms of computing requirements.

**Figure 5:**
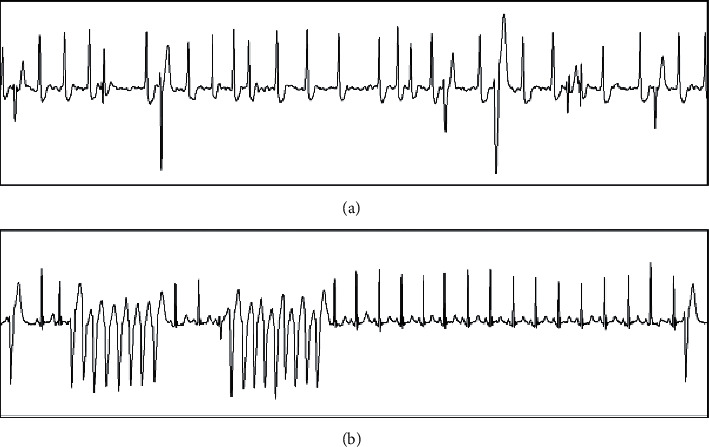
Two groups of ECG diagrams (203 and 205). (a) MIT-BIH ECG data no. 203. (b) MIT-BIH ECG data no. 205.

In addition, it can be seen from Figure 5 that this algorithm shows a strong anti-interference capability in the detection of moving plate experiment and can effectively reduce the ECG noise and baseline drift brought by movement, so that it can effectively detect and monitor the ECG in different motion states. At the same time, the operation speed and resource share of the algorithm can guarantee the real-time and durability of ECG monitoring.

It is important to note that the algorithm of R-wave detection is faulty in some occasions, such as MIT-BIH database in 203 and 205 of two groups of ECG data waveform, the waveform memory is intermittent R-wave inversion, the phenomenon of greater influence on the sensitivity of the proposed algorithm, leads to recognition of the R-wave in 203 and 205 groups of data, and the sensitivity of 99.00% and 98.04%, as shown in Figure 5.

In summary, R-wave detection was evaluated on the existing standard MIT-BIH database. The algorithm has a relatively high performance, with 99.70% sensitivity and 99.93% positive predictability, showing obvious advantages. In addition, its low computational requirements and good anti-interference capability make it easy to deploy and implement in portable monitoring and motion monitoring applications. We will further study the limitations of the current algorithm in order to develop a more complete and practical algorithm.

## Figures and Tables

**Table 1:** Exercise test results.

Number of samples	Total time of exercise (s)	Maximum heart rate (bmp)	The total number of R-waves	Number of leaks or errors	Accuracy (%)
10	3922	168 ± 2	8619	2	99.98

**Table 2:** MIT-BIH detection R-wave results.

Number of samples	The total number of R-waves	TP	FN	FP	Se (%)	+P (%)
21	47498	47398	143	31	99.70	99.93

**Table 3:** Comparison with other methods' test results.

Method	Se (%)	+*P*(%)
Pandit et al. [2]	99.62	99.67
Lai et al. [24]	99.69	99.63
Method in this paper	99.70	99.93

## Data Availability

The ECG data used to support the findings of this study have been deposited in the MIT-BIT repository.
